# Assessing the suitability of general practice electronic health records for clinical prediction model development: a data quality assessment

**DOI:** 10.1186/s12911-021-01669-6

**Published:** 2021-10-30

**Authors:** Sharmala Thuraisingam, Patty Chondros, Michelle M. Dowsey, Tim Spelman, Stephanie Garies, Peter F. Choong, Jane Gunn, Jo-Anne Manski-Nankervis

**Affiliations:** 1 Department of Surgery, University of Melbourne, 29 Regent Street, Fitzroy, VIC 3065 Australia; 2 Department of General Practice, University of Melbourne, 780 Elizabeth Street, Parkville, VIC 3010 Australia; 3 Karolinska Institute, Solnavagen 1, 171 77 Solna, Sweden; 4 Faculty of Medicine Dentistry & Health Sciences, University of Melbourne, Alan Gilbert Building, Level 2, Carlton, VIC 3053 Australia; 5 Department of Family Medicine, Cumming School of Medicine, University of Calgary, Alberta, T2N 4N1 Canada

**Keywords:** Electronic medical records, Electronic health records, Data quality assessment, General practice, Primary care, Clinical prediction model development, Data linkage

## Abstract

**Background:**

The use of general practice electronic health records (EHRs) for research purposes is in its infancy in Australia. Given these data were collected for clinical purposes, questions remain around data quality and whether these data are suitable for use in prediction model development. In this study we assess the quality of data recorded in 201,462 patient EHRs from 483 Australian general practices to determine its usefulness in the development of a clinical prediction model for total knee replacement (TKR) surgery in patients with osteoarthritis (OA).

**Methods:**

Variables to be used in model development were assessed for completeness and plausibility. Accuracy for the outcome and competing risk were assessed through record level linkage with two gold standard national registries, Australian Orthopaedic Association National Joint Replacement Registry (AOANJRR) and National Death Index (NDI). The validity of the EHR data was tested using participant characteristics from the 2014–15 Australian National Health Survey (NHS).

**Results:**

There were substantial missing data for body mass index and weight gain between early adulthood and middle age. TKR and death were recorded with good accuracy, however, year of TKR, year of death and side of TKR were poorly recorded. Patient characteristics recorded in the EHR were comparable to participant characteristics from the NHS, except for OA medication and metastatic solid tumour.

**Conclusions:**

In this study, data relating to the outcome, competing risk and two predictors were unfit for prediction model development. This study highlights the need for more accurate and complete recording of patient data within EHRs if these data are to be used to develop clinical prediction models. Data linkage with other gold standard data sets/registries may in the meantime help overcome some of the current data quality challenges in general practice EHRs when developing prediction models.

**Supplementary Information:**

The online version contains supplementary material available at 10.1186/s12911-021-01669-6.

## Background

General practice electronic health records (EHRs), primarily used to document clinical care, are a rich source of patient data. They contain information on patient socio-demographic characteristics, medical history, pathology results, prescriptions, immunisations, clinical observations and billing details. There have been significant developments in general practice EHR software systems over time, resulting in increases in the volume, detail and quality of patient data that can be stored within these records [1]. As a result of this, and technological advancements in computer processing power, general practice EHRs have the potential to answer various research questions [2].

Despite the potential to answer a variety of research questions, the large volumes of data within EHRs does not necessarily mean these data are fit to answer the research question [2]. EHRs are a secondary data source collected for clinical purposes and not research. Therefore, the quality of the data in EHRs may be influenced by the methods and practices used to record, extract, collate and disseminate the data [3–5]. For example, chronic conditions that are incentivised or that are national health priorities, such as asthma may be more completely recorded in the EHR [2]. Therefore, the prevalence of these conditions may be more accurately recorded in EHRs than other unincentivised or lower priority conditions.

In addition, there is no standardisation of general practice EHR software in Australia and no national standards for EHR software [6]. Each EHR software system differs in the clinical terminologies and classifications used and many still utilise text heavy fields for data capture [6, 7]. Hence the quality of data in EHRs may be influenced by the design and layout of the software system used to collect patient information.

Kahn et al. (2016) devised a framework for assessing the quality of data within EHRs to assist researchers in determining whether their data are fit to answer the research question. In this study we utilised this framework to assess whether the quality of data in a sample of Australian general practice EHRs were suitable for use in the development of a clinical prediction tool for total knee replacement (TKR) in patients with osteoarthritis (OA) for use in primary care. Thirty-two predictors were identified through a literature review and by consultation with experts in the field of OA [8]. Nine of these predictors were available in general practice EHRs and therefore selected for use in model development. The planned outcome of the model was time to primary TKR, with death treated as a competing risk due to the age of the study cohort. The specific methods used to develop the prediction tool are detailed in Thuraisingam et al. [8] and are outside the scope of this study. Here we assessed the quality of the EHR data prior to model development. Data were verified through linkage with gold standard registries, and validity through comparison with national data. The data linkage process was assessed to identify potential bias that may have been introduced from data linkage.

## Methods

### Study design

NPS MedicineWise manage the MedicineInsight data set consisting of deidentified EHRs from 2.9 million patients from 671 general practices across Australia [9, 10]. These data are provided by consenting practices and are extracted from two different EHR software systems using two third-party data extraction tools [10]. Data from the two EHR software systems are amalgamated within the data warehouse into a single consistent structure [10]. The coding used to merge data fields is proprietary of NPS MedicineWise and has been developed with input from general practitioners, pharmacists, business analysts and data warehouse architects. For this study, NPS MedicineWise provided EHR data extracted from 475,870 patients with a recorded diagnosis of OA (see Additional file 1: Table 1 for coding of OA). These records included patient clinical data recorded in the EHR at the 31^st^ of December 2017. Patient encounter data were provided for the years 2013 to 2017.

These EHR data were linked with Australian Orthopaedic Association National Joint Replacement Registry (AOANJRR) [11] and the National Death Index (NDI) [12]. The AOANJRR contains data on TKRs performed in Australia since 1st of September 1999 with near complete capture of all TKRs in Australia from 2002 onwards. The NDI includes all deaths that have occurred in Australia since 1999. The data linkage process is outlined in Additional files 2–4.

Study baseline was the 1st of January 2014 and the study end date the 31st of December 2017 (inclusive). The inclusion criteria for the study were patients: (i) with at least two visits to the clinic in the year prior to baseline (i.e. in 2013); (ii) aged 45 years and over at baseline; (iii) alive at study baseline (i.e. no record of death in the NDI) and (iv) no recorded evidence of bilateral TKR prior to study baseline. We were unable to determine active patients of a clinic according to the RACGP definition (at least three clinic visits in a two-year period) given the encounter data provided did not include the two years prior to study baseline [13]. Hence criterion (i) was chosen as a proxy.

### Coding of variables

The nine candidate predictors were age, body mass index (BMI), weight gain between early adulthood and middle age, prescribing of OA medication in the year prior to baseline, multimorbidity count, diagnosis of a mental health condition, previous contralateral TKR, other knee surgery (excluding TKR) and geographical residence of the patient. Each of the predictors were coded from the EHR at study baseline except for BMI and the prescribing of OA medications. The last BMI measurement recorded in the EHR in the year prior to the start of the study was included. The strength, dosage and frequency fields for medications data were used to determine whether patients were likely to be taking medications for OA at study baseline using prescriptions issued in the 12 months prior to baseline. Death was coded from the patient status variable in the EHR. The patient’s geographical residence was based on the Australian Bureau of Statistics (ABS) Australian Statistical Geography Standard (ASGS) remoteness areas [14].

Multimorbidity count was used as a proxy measure for overall health. Three different ways of counting multimorbidity were considered: (i) count of chronic conditions listed in the Charlson Comorbidity Index (CCI) which predicts ten year survival in patients with multiple comorbidities [15], (ii) count of 17 frequently managed chronic conditions in primary care as identified in the Bettering the Evaluation and Care of Health (BEACH) study [16], and (iii) a combination of (i) and (ii). The conditions included in the CCI and from the BEACH study are listed in Additional file 1 Table 2, and coding in Table 3. Coding for mental health conditions, past knee surgeries and medications for osteoarthritis are detailed in Additional file 1 Tables 4–6 respectively.

Patients were coded as having missing data for the prescribing of OA medications if they had a prescription issued 12 months prior to baseline with missing dosage, strength or frequency and no other OA medication prescription with a clear end date, as it was not possible to determine whether the patient was likely to be taking medications for OA at study baseline. Similarly, patients were coded as having missing data for chronic conditions and past surgeries if they had missing diagnoses and surgery dates since it was not possible to determine whether the condition was present, or surgery had occurred by study baseline. Patients that did not have a text entry in the diagnosis field relating to any of the chronic conditions in Additional file 1 Table 2, were coded as negative for that condition and those without a prescription entry for the medications listed in Additional file 1 Table 6 were coded as negative for OA medications. Patients without a BMI measurement recorded in the year prior to baseline were coded as having missing values for BMI. The same approach was used for weight. Those with a recorded patient status of “deceased” with no year of death recorded were coded as having missing year of death.

### Data quality assessment

The data quality assessment of the MedicineInsight EHR data included the following steps:

(i) Identification of missing and implausible data
(ii) Assessment of accuracy of recording of TKR (outcome) and death (competing risk) in the EHR
(iii) Assessment of external validity of EHR data

The methods used in steps 2(i), 2(ii) and 2(iii) are detailed below.

(i) *Identification of missing and implausible data*
  The completeness and plausibility of the predictors, outcome and competing risk were assessed. Counts and percentages were used to summarise the amounts of missing data and implausible values. Definitions for implausible data entries are listed in Additional file 5. Examples include year of birth documented as a date after the data extraction date and year of death being documented before the year of birth.
(ii) *Assessment of accuracy of recording of TKR and death in the EHR*
  The accuracy, sensitivity and specificity of recording of TKR and death in the MedicineInsight EHR data set were assessed through data linkage with the AOANJRR and NDI respectively. Sensitivity, specificity and accuracy were calculated using the definitions in Altman and Bland (1994). In assessing the accuracy of recording of TKR side and year of surgery, the denominator was the total number of true TKRs regardless of whether a side or year was recorded. For model building purposes we are interested in the proportion of true TKRs that had a side and year of surgery correctly recorded, not the proportion of recorded TKR sides and years that were correctly recorded. The same approach was used to assess the accuracy of recording of year of death. The data linkage process used to link the NPS MedicineWise EHR data set to the AOANJRR and NDI was assessed using the checklist developed by Pratt et al. [17].
(iii) *Assessment of external validity of EHR data*
  The external validity of the EHR data set was assessed by comparing socio-demographics and clinical characteristics of our cohort with that of OA patients aged 45 years and over from the 2014–2015 National Health Survey (NHS) [18] carried out by the ABS. The ABS NHS condition level codes [19] used to code the various chronic conditions are listed in Additional file 6. Not all chronic conditions included in our multimorbidity measures were available in the NHS, hence we compared the most commonly occurring chronic conditions for patients with OA as determined by the Australian Institute of Health and Welfare (AIHW) [20]. Proportions from the NHS data set were adjusted to account for the survey sampling strategy. The participant household record identifier was defined as the primary sampling unit in the NHS data and standard errors estimated using replicate weights and the jackknife variance estimator [21]. Due to the NHS sampling method, counts have not been provided for these data. Instead, estimated population proportions and standard errors for these proportions have been calculated using the methods outlined in Donath (2005). For the EHR data, the general practice clinic was used as the primary sampling unit.

### Statistical analyses

Categorical variables were summarised using frequency and percentage. Continuous variables were summarised using mean and standard deviation (SD) or median and inter-quartile range (IQR) as appropriate. All analyses were conducted using STATA MP version 16.1 (StataCorp, College Station Texas) [22].

## Results

### Selection of study cohort

**Fig. 1:**
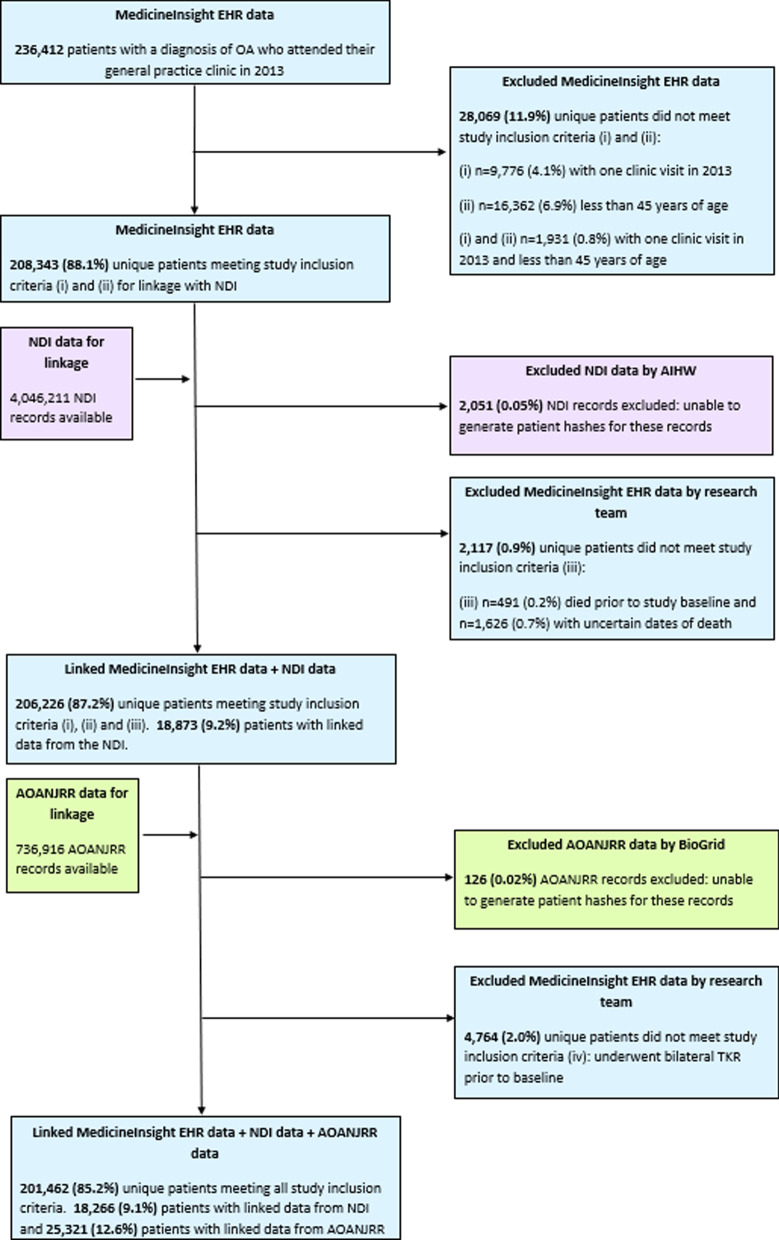
Selection of study cohort from EHRs. Note: percentages in this diagram may not sum to 100% due to rounding

Of the 475,870 patient EHRs, 236,412 patients with a recorded diagnosis of OA prior to study baseline who attended their general practice clinic in the year prior to baseline were identified (Fig. 1). A total of 34,950 (14.8%) patients were excluded from the study. Approximately 28,069 (11.9%) were excluded due to (i) less than two visits to the clinic in the year prior to baseline (n = 9776), (ii) less than 45 years of age (n = 16,362) or (iii) both (i) and (ii) (n = 1931). After linkage with the NDI, a further 0.9% (n = 2117) of the 236,412 patients were excluded because they were either not alive at study baseline (n = 491, 0.2%) or they could not be confirmed as being alive (n = 1626, 0.7%) due to uncertain dates of death recorded in the NDI. Uncertain dates of death were due to patients with common names and dates of birth having links to multiple records in the NDI and hence multiple possible dates of death, deaths being discovered some time after the event or missing date of death data. After linkage with the AOANJRR, 4,764 patients (2.0%) had undergone bilateral TKR prior to study baseline and therefore no longer considered at risk of the outcome (i.e. primary TKR) during the study period. A total of 201,462 (85.2%) patients from 483 general practices across Australia fully met all study inclusion criteria, with 9% (n = 18,266) having a linked record from the NDI and 12.6% (n = 25,321) having a linked record from the AOANJRR. During the data linkage process, 0.05% of records available from NDI and 0.02% of records available from AOANJRR were excluded by the respective linkage providers due to issues generating patient linkage keys (hashes) for linkage.

### Data quality assessment

(i) *Missing and implausible data*
  Missing data and implausible values for the predictors, outcome and competing risk are presented in Table 1. Missing data for socio-demographic characteristics, age and patient geographical residence were minimal. However, there were substantial missing data for BMI (68%) in the year prior to baseline, and weight gain between early adulthood and middle age was unable to be calculated as none of the patients had weight during early adulthood recorded in their EHR. Year of death was missing in the EHR for approximately 19% of patients whose status was recorded as “deceased”.
  Approximately 16% of patients had at least one prescription with a missing dosage, strength, or frequency for an osteoarthritis medication. For the chronic conditions included in the BEACH count and combined CCI and BEACH count, approximately 25% of patients had at least one diagnoses date missing. Of the patients with a TKR recorded in the study period, over 90% did not have the side of TKR recorded. Overall, implausible data entries, according to the definitions in Additional file 5 were minimal.

**Table 1:** Missing data and implausible values in EHRs for model predictors and outcomes

	Study cohort (N = 201,462)	Missing data	Implausible data entries
Characteristics	n (%)	n (%)	n (%)
*Predictors*			
Age (years)—mean (SD)	67.2 (11.1)	3 (0.001)	–
BMI—mean (SD)	30.1 (6.5)	137,071 (68.0)	224 (0.1)
Weight gain between early adulthood and middle age—mean (SD)	–	201,462 (100.0)	–
Weight early adulthood—mean (SD)	–	201,462 (100.0)	–
Weight middle age—mean (SD)	85.3 (20.1)	113,561 (56.4)	135 (0.07)
OA medication prescription/s	57,090 (33.8)	32,548 (16.2)	43 (0.02)
Multimorbidity			
CCI count—Median (IQR)	0 [0,1]	15,875 (7.9)	N/A
BEACH count^—Median (IQR)	2 [1, 4]	51,035 (25.3)	N/A
Combined CCI and BEACH count^—Median (IQR)	2 [1, 4]	51,876 (25.7)	N/A
Mental health condition	46,859 (24.2)	8178 (4.1)	N/A
Previous/contralateral knee replacement	9432 (4.7)	1254 (0.6)	10 (0.005)
Any past knee surgery on either knee (excluding TKR)	6070 (3.0)	990 (0.5)	17 (0.01)
Patient geographical residence*		1068 (0.5)	N/A
Major cities of Australia	111,075 (55.4)		
Inner regional Australia	62,221 (31.1)		
Remote Australia	27,098 (13.5)		
*Outcome*			
TKR during study	8638 (4.3)	1259 (0.6)	7 (0.003)
TKR year		1259 (0.6)	7 (0.003)
2014	2282 (26.4)		
2015	2186 (25.3)		
2016	2171 (25.1)		
2017	1999 (23.1)		
TKR side		9134 (92.2)	
Left	326 (42.3)		
Right	358 (46.5)		
Bilateral	86 (11.2)		
*Competing risk*			
Recorded as “deceased” in EHR	9581 (4.8)	–	–
Recorded year of death of those recorded as deceased in the EHR	7720 (80.6)	1861 (19.4)	–
Recorded as prior to baseline	271 (3.5)		
2014	1445 (18.7)		
2015	1866 (24.2)		
2016	2060 (26.7)		
2017	2078 (26.9)		
*General practice clinics (n* = *483)*			
Median (IQR) number of patients with OA per clinic	327 [181, 566]	–	–
Clinic state		–	–
New South Wales	71,397 (35.4)		
Queensland	38,498 (19.1)		
Victoria	36,189 (18.0)		
Western Australia	22,570 (11.2)		
Tasmania	21,247 (10.6)		
South Australia	6018 (3.0)		
Australian Capital Territory	4168 (2.1)		
Northern Territory	1375 (0.7)		
Clinic geographical location*	71 (0.04)		
Major cities of Australia	114,203 (56.7)		
Inner regional Australia	61,501 (30.5)		
Remote Australia	25,687 (12.8)		

Counts and percentages presented unless otherwise indicated. Percentages may not add to 100% due to rounding

*BMI* body mass index, *OA* osteoarthritis, *CCI* Charlson Comorbidity Index, *IQR* Inter-Quartile Range, *BEACH* Bettering the Evaluation and Care of Health, *TKR* total knee replacement, *N*/*A* Not applicable

^excluding mental health conditions

^*^Based on the Australian Bureau of Statistics (ABS) Australian Statistical Geography Standard (ASGS) remoteness areas [14]

Notes: BMI includes measurements recorded within one year of study baseline; Early adulthood = 18–21 years; Middle age = 45–65 years; Patient considered to be on OA medication if estimated to be on medication at study baseline using prescription date and medication strength, dosage and frequency; Patient considered to have chronic condition or undergone past knee surgery if record of this exists prior to study baseline
(ii) *Accuracy of recording of TKR and death data*
  TKR and death were recorded with good accuracy in the EHR, approximately 97% and 96% respectively (Table 2). Approximately 57% of true TKRs had a correctly recorded year of surgery and 49% of true deaths a correctly recorded year of death. A small proportion of true TKRs had the side correctly recorded (3.4%).
  Results from the assessment of the data linkage process are detailed in Additional files 7, 8. Only a small proportion of patients (1.6%) were excluded during the data linkage process due to either uncertain dates of death in the NDI or the inability to generate linkage keys to link these patients with the EHR data. Hence, it seems unlikely that bias was introduced from the data linkage process.

**Table 2:** Accuracy of recording of TKR and death in EHRs

N = 201,462	TKR	Death
True positives	7854	6304
True negatives	186,937	187,527
False positives	784	3277
False negatives	4621	4354
Sensitivity	63.0%	59.1%
Specificity	99.6%	98.3%
Accuracy	96.7%	96.2%
Proportion of true TKRs with a correctly recorded year of surgery	57.2%	–
Proportion of true TKRs with a correctly recorded side	3.4%	–
Proportion of true deaths with a correctly recorded year of death	–	49.0%

AOANJRR considered gold-standard for TKR data and NDI for death data; “-” represents “N/A”
(iii) *External validity of the EHR data*
  Characteristics of patients with OA aged 45 years and over were compared between the EHR data and the 2014–15 NHS data (Table 3). Socio-demographic and clinical characteristics were similar between the two cohorts except for proportions relating to OA medication (EHR 34% vs NHS 55%) and metastatic solid tumour (EHR 17% vs NHS 26%).

**Table 3:** Summary statistics of patient characteristics from EHR data and ABS NHS data

	EHR data (N = 201,462)			ABS National Health Survey 2014–15 (N = 2,069,060)	
	n	%	95% CI	%	95% CI
*Socio-demographics* Age category (years)					
45–49	11,477	5.7	5.4 to 6.0	5.6	4.3 to 6.9
50–54	18,453	9.2	8.8 to 9.5	9.1	7.3 to 10.9
55–59	24,102	12.0	11.6 to 12.3	13.1	11.2 to 15.0
60–64	29,745	14.8	14.5 to 15.1	17.6	15.9 to 19.3
65–69	33,172	16.5	16.2 to 16.8	17.8	16.0 to 19.6
70–74	27,902	13.8	13.6 to 14.1	13.5	11.7 to 15.3
75–79	23,078	11.5	11.2 to 11.8	10.0	8.5 to 11.5
80 +	33,530	16.6	16.0 to 17.3	13.2	11.2 to 15.2
Sex					
Male	78,049	38.7	38.1 to 39.3	35.6	33.3 to 37.9
Female	123,376	61.2	60.6 to 61.8	64.4	62.1 to 66.7
Other	37	0.02	0.01 to 0.03	-	-
Remoteness area					
Major cities of Australia	111,075	55.4	49.9 to 60.9	63.6	60.7 to 66.4
Inner regional Australia	62,221	31.1	26.2 to 36.3	23.3	20.1 to 26.6
Remote Australia	27,098	13.5	10.5 to 17.2	13.1	10.7 to 15.5
*Clinical characteristics*					
BMI, *mean (SD)*	30.1 (6.5)	N/A	N/A	29.3 (8.8)	N/A
OA medication prescription/s	57,090	33.8	32.9 to 34.7	54.9	52.1 to 57.7
Chronic conditions*					
Hypertension	81,004	44.4	43.5 to 45.3	40.0	37.0 to 42.9
Lipid disorder	58,478	31.2	30.1 to 32.3	26.6	24.1 to 29.1
Ischaemic heart disease (IHD)	23,522	11.8	11.4 to 12.2	6.8	5.2 to 8.3
Asthma	21,757	11.2	10.7 to 11.7	17.2	14.7 to 19.8
Diabetes	27,821	14.1	13.7 to 14.6	15.1	12.9 to 17.3
Chronic obstructive pulmonary disease (COPD)	12,709	6.4	6.0 to 6.8	8.8	7.5 to 10.1
Metastatic solid tumour	33,485	16.9	16.3 to 17.5	26.3	24.2 to 28.4
Depression	35,644	19.6	19.0 to 20.2	11.9	10.1 to 13.7
Anxiety and other neurotic, stress related and somatoform disorders	19,393	11.7	11.2 to 12.2	15.4	13.2 to 17.6

*CI*  confidence interval

^*^ most commonly occurring chronic conditions as identified by AIHW [20]

Note: Estimates from the NHS have been calculated using replicate weights and the jacknife variance estimator [21]

## Discussion

### Are these data fit for use?

In this data quality assessment, we considered the completeness, plausibility, accuracy and validity of data contained in general practice EHRs, specifically for the purpose of developing a prediction model from these data. We found data fields relating to the outcome and competing risk (TKR side, TKR year and year of death) to be incomplete and inaccurate and therefore unfit for use in model development. The predictors BMI and weight gain between early adulthood and middle age were also unfit for use due to high proportions of missing data.

The remaining predictors had less than 35% missing data or implausible values, which would allow us to perform multiple imputation to impute missing predictor values prior to model development as outlined in our published statistical analysis plan [8], provided we include variables to explain the missing data. We were unable to assess the accuracy and external validity of the candidate predictors due to restricted access to other data sets containing this information. We therefore cannot be certain that these predictors were accurately recorded in the EHRs. However, the socio-demographic and clinical characteristics of our cohort and the NHS cohort were similar, except for the prescribing of OA medication and recording of metastatic solid tumour. The NHS data had a higher proportion of OA medication prescribed compared to the EHR and a likely explanation is that the NHS data included over the counter medications as well as medications prescribed by specialists which might not be communicated to the general practitioner. The lower proportion of metastatic solid tumours in the EHR data compared to the NHS data may be due to diagnoses by specialists not being communicated to the general practitioner and the inconsistent manner in which metastatic solid tumours are recorded between general practitioners.

Whilst it seems important to assess the accuracy and validity of all predictors prior to model development, if we consider the context in which the model will be used, it may not be necessary to validate the predictors outside of the EHR setting. Our intention is to embed the prediction model in a clinical support decision tool within the EHR such that the predictors are drawn directly from the record. The main aim of the model is to produce accurate predictions, hence provided these data are complete/near complete, the predictors may only need to be representative of data within the EHRs. This viewpoint suggests that the predictors (excluding BMI and weight gain) in our study may be fit for our purpose. Should these predictors be used for model development (with outcome data obtained through data linkage) and recording practices in EHRs change over time, the model may not perform well and will need to be updated periodically with new data from the EHRs.

### Strengths and limitations

Our study adds to the limited literature on the quality of data within Australian general practice EHRs. It is the first Australian study to provide insight into the use of these data specifically for prediction models through the development of a real-world clinical prediction tool for use in practice. Our study highlights the importance of assessing the suitability of EHR data prior to model development through data quality assessment and demonstrates how to conduct such a study. It provides insight into which data fields in Australian EHRs are prone to missing and inaccurate data and the value of data linkage for data validation.

In this study we followed established guidelines for assessing data quality and the data linkage process [17, 23]. Our assessment was based on a large sample of EHRs which provides a true representation of how data are recorded in general practice EHRs. Our coding of diagnoses were consistent with NPS MedicineWise MedicineInsight Data Book [9]. Lastly, the data quality assessment included input from general practitioners, epidemiologists and biostatisticians.

Whilst the NDI data are validated annually against the Australian mortality data [24], the results are not publicly published and it is possible that there may be some uncertainty in these data. Although the AOANJRR has near complete capture of every joint replacement performed in Australia and good external validity of these data has been demonstrated [11], it is possible that some patients within our data set underwent TKR in another country.

The EHRs provided by NPS MedicineWise to our research team consisted of patients identified as having a recorded diagnosis of OA in their EHR. The selection of this cohort was performed by NPS MedicineWise and we were provided the free-text terms used to identify the cohort. Whilst it is possible that data cleaning errors may have occurred during data pre-processing, the proportion of patient EHRs with a recorded diagnosis of OA provided to us out of the total number of patients was approximately 10.2% (304,725/2,974,031). This is comparable to the estimate provided by AIHW of 9.3% of Australians living with OA in 2017–18 [20]. Similarly, the rate of TKR in our cohort (229 per 100,000 per year) obtained from linkage with AOANJRR was similar to that published by AIHW (218 per 100,000 per year) [20]. We were unable to verify the rate of death obtained through linkage with the NDI as we were unable to find another data source containing this information. Therefore, it is possible that the observed death rate is different to the expected death rate and our assessment of accuracy of recording of death is inaccurate. However, this seems unlikely as only a small proportion of patients were excluded from our study for uncertain dates of death in the NDI (0.7%) and a small proportion of records from the NDI (0.05%) were excluded as hashes for linkage could not be generated. Further, data linkage errors such as errors generating patient hashes or matching hashes is expected to be uncommon and have little impact on our assessment of accuracy.

This study identified 201,462 patients with OA from 483 general practices across Australia. It is possible that duplicate patients exist within our cohort given patients are not registered to one general practice clinic in Australia and are able to attend multiple clinics. The MedicineInsight General Practice Insights Report from 2017–2018 estimates that approximately 3% of patients in the 2017–2018 MedicineInsight cohort are duplicate patients [25]. It is therefore plausible to assume that the proportion of duplicated patients in our cohort is small.

Further, given there is no global measure for overall health we have considered using a count of conditions from the CCI [15] and a count of chronic conditions that were identified from the BEACH study as being frequently managed in general practice [16]. The latter is not a validated measure and may not accurately represent a patient’s overall health status. All data fields used in this study were provided as raw text fields except for age, gender, patient status (active, inactive or deceased), geographical location of the patient and clinic state. Data extracted for these variables from the two EHR software systems were merged into a common variable within the data warehouse. There were minimal missing data for these merged data fields. Patient status is recorded similarly in the two EHR software systems and therefore inaccuracies in the recording of deceased patients were likely due to clinics not being informed of the death of a patient or data entry errors, rather than the merging of these data. Given the majority of data fields were text, extensive data cleaning was carried out by our research team on prescriptions and diagnoses data fields to extract the required information, and data sets were extensively reshaped and merged to prepare the data into the format required for assessment and later, modelling. Whilst we were able to externally validate some of the predictors, we cannot be certain bias was not introduced from data pre-processing errors.

Lastly, we were not provided data from the “progress notes” text field within the EHR to ensure patient privacy was maintained. It is possible that important patient information such as BMI were recorded in this field as opposed to the field allocated specifically for clinical observations. This highlights the need for regulated EHR recording practices and enforced EHR software standards across Australia. Without this, Australia will continue to fall behind other developed nations in its use of health information [6, 26].

## Conclusions

The use of general practice EHRs for clinical prediction model development is in its infancy in Australia. In this study, data relating to the outcome, competing risk and two predictors were unfit for prediction model development. This study highlights the importance of conducting thorough data quality assessments prior to model development to assess the suitability of the EHR data. These assessments need to extend beyond missing and implausible data and include assessments of accuracy. External validity can provide useful insights about the study cohort and is important for models being applied outside the EHR setting. There is a need for more accurate and complete recording of patient data within EHRs if these data are to be used to develop clinical prediction models. Data linkage with other gold standard data sets/registries may in the meantime help overcome some of the current data quality challenges in general practice EHRs when developing prediction models.

## Supplementary Information

**Additional file 1**: Coding of variables.

**Additional file 2**: Data linkage methodology.

**Additional file 3**: Methodology for linkage with AOANJRR.

**Additional file 4**: Methodology for linkage with NDI.

**Additional file 5**: Implausible data entry definitions.

**Additional file 6**: Coding of Australian Bureau of Statistics National Health Survey Chronic Conditions.

**Additional file 7**: Assessment of the data linkage process.

**Additional file 8**: Comparison of patient characteristics from EHRs linked with NDI and EHRs excluded due to uncertain dates of death in NDI.

## Data Availability

The data that support the findings of this study are available from NPS MedicineWise, Australian Orthopaedic Association, Australian Institute of Health and Welfare and the Australian Bureau of Statistics but restrictions apply to the availability of these data, which were used under license for the current study, and so are not publicly available. Data may be available from the authors upon reasonable request if permission is granted by the parties listed above. The data sets provided by NPS MedicineWise, Australian Orthopaedic Association and Australian Institute of Health and Welfare are not publicly available due to patient privacy. National Health Survey data may be available upon reasonable request with permission of the Australian Bureau of Statistics.

## Abbreviations

ABS: Australian Bureau of Statistics
AIHW: Australian Institute of Health and Welfare
AOANJRR: Australian Orthopaedic Association National Joint Replacement Registry
ASGS: Australian Statistical Geography Standard
BEACH: Bettering the Evaluation and Care of Health
BMI: Body mass index
CCI: Charlson comorbidity index
COPD: Chronic obstructive pulmonary disease
DQA: Data quality assessment
EHR: Electronic health record
IHD: Ischaemic heart disease
IQR: Interquartile range
MP: Multiprocessor
NDI: National Death Index
NHS: National Health Survey
NPS: National Prescribing Service
OA: Osteoarthritis
RACGP: Royal Australian College of General Practitioners
SD: Standard deviation
TKR: Total knee replacement
USI: Unique subject identifier
