# Supplementary material for: Assessing the suitability of general practice electronic health records for clinical prediction model development: a data quality assessment
Source: BMC Med Inform Decis Mak. 2021 Oct 30;21:297. doi: 10.1186/s12911-021-01669-6 (PMC8557028; doi:10.1186/s12911-021-01669-6)
Supplement: Supplementary file 1 — Additional file 1: Coding of variables. [file 12911_2021_1669_MOESM1_ESM.docx]

# Additional file 1. Coding of variables

| **TABLE 1. CODING OF OSTEOARTHRITIS BY NPS MEDICINEWISE** | |
| --- | --- |
| **CONDITION** | **CODED FROM EHR DIAGNOSIS (DROP-DOWN) MENU** |
| OSTEOARTHRITIS | ANKLE OSTEOARTHRITIS |
|  | ARTHRITIS – OSTEO |
|  | CERVICAL – OSTEO ARTHRITIS |
|  | CERVICAL SPINE OSTEOARTHRITIS |
|  | ELBOW OSTEOARTHRITIS |
|  | GENERALISED OSTEOARTHRITIS |
|  | HALLUX RIGIDUS |
|  | HIP OSTEOARTHRITIS |
|  | HIP OSTEOARTHROSIS |
|  | KNEE OSTEOARTHRITIS |
|  | KNEE OSTEOARTHROSIS |
|  | LUMBAR – DISC DISEASE |
|  | LUMBAR – OSTEO ARTHRITIS |
|  | LUMBAR SPINE OSTEOARTHRITIS |
|  | LUMBO-SACRAL DISC DISEASE |
|  | MIDFOOT OSTEOARTHRITIS |
|  | OA |
|  | OA (OSTEOARTHRITIS) |
|  | OSTEOARTHRITIS |
|  | OSTEOARTHRITIS – ANKLE |
|  | OSTEOARTHRITIS – ELBOW |
|  | OSTEOARTHRITIS – FINGERS |
|  | OSTEOARTHRITIS – GLENOHUMERAL JOINT |
|  | OSTEOARTHRITIS – HANDS |
|  | OSTEOARTHRITIS – HIP |
|  | OSTEOARTHRITIS – KNEE |
|  | OSTEOARTHRITIS – NECK |
|  | OSTEOARTHRITIS – SHOULDER |
|  | OSTEOARTHRITIS – SPINE |
|  | OSTEOARTHRITIS OF 1^ST^ CARPOMETACARPAL JOINT |
|  | OSTEOARTHRITIS OF 1^ST^ CARPO-METACARPAL JOINT |
|  | OSTEOARTHRITIS OF 1^ST^ METATARSOPHALANGEAL JOINT |
|  | OSTEOARTHRITIS OF ANKLE |
|  | OSTEOARTHRITIS OF CERVICAL SPINE |
|  | OSTEOARTHRITIS OF ELBOW |
|  | OSTEOARTHRITIS OF FINGERS |
|  | OSTEOARTHRITIS OF FOOT |
|  | OSTEOARTHRITIS OF HAND |
|  | OSTEOARTHRITIS OF HIP |
|  | OSTEOARTHRITIS OF KNEE |
|  | OSTEOARTHRITIS OF LUMBAR SPINE |
|  | OSTEOARTHRITIS OF NECK |
|  | OSTEOARTHRITIS OF SACROILIAC JOINTS |
|  | OSTEOARTHRITIS OF SHOULDER |
|  | OSTEOARTHRITIS OF THE PATELLOFEMORAL JOINT |
|  | OSTEOARTHRITIS OF THORACIC SPINE |
|  | OSTEOARTHRITIS OF TMJ |
|  | OSTEOARTHRITIS OF WRIST |
|  | OSTEOARTHRITIS, GENERALISED |
|  | OSTEOARTHROSIS |
|  | PATELLOFEMORAL OSTEOARTHRITIS |
|  | SACROILIAC JOINT ARTHRITIS |
|  | SHOULDER OSTEOARTHRITIS |
|  | SPONDYLOSIS |
|  | THORACIC – OSTEO ARTHRITIS |
|  | WEAR AND TEAR ARTHRITIS |
|  | WRIST OSTEOARTHRITIS |
|  | **FREE TEXT TERMS SEARCHED IN THE EHR ‘DIAGNOSIS’, ‘REASON FOR VISIT’ AND ‘REASON FOR PRESCRIPTION’ FIELDS** |
|  | ARTHRITIS – OSTEO |
|  | CERVICAL – OSTEO ARTHRITIS |
|  | HALLUX RIGIDUS |
|  | LUMBAR – DISC DISEASE |
|  | LUMBAR – OSTEO ARTHRITIS |
|  | LUMBO-SACRAL DISC DISEASE |
|  | OA |
|  | SACROILIAC JOINT ARTHRITIS |
|  | SPONDYLOSIS |
|  | THORACIC – OSTEO ARTHRITIS |
|  | WEAR AND TEAR ARTHRITIS |

| **Table 2. List of chronic conditions included in Charlson Comorbidity Index (CCI)** |
| --- |
| Myocardial infarction (MI) |
| Congestive heart failure (CHF) |
| Peripheral vascular disease (PVD) |
| Cerebrovascular accident (CVA)/transient ischaemic attack (TIA) |
| Dementia |
| Chronic obstructive pulmonary disease (COPD) |
| Connective tissue disease |
| Peptic ulcer disease |
| Liver disease |
| Diabetes mellitus |
| Hemiplegia |
| Chronic kidney disease (CKD) |
| Leukaemia |
| Lymphoma |
| Malignant tumour |
| Human immunodeficiency virus (HIV)/Acquired immunodeficiency syndrome (AIDs) |
| **List of chronic conditions most frequently managed in primary care from BEACH study included in BEACH comorbidity count** |
| Hypertension |
| Diabetes mellitus |
| Lipid disorder |
| Dyspepsia |
| Asthma |
| Atrial fibrillation (AF) |
| Malignant neoplasms of the skin |
| Osteoporosis |
| Hypothyroidism/myxoedema |
| Ischaemic heart disease (IHD) |
| Chronic obstructive pulmonary disease (COPD) |
| Gout |
| Migraine |
| Congestive heart failure (CHF) |
| Chronic pain not otherwise specified |
| Dementia |
| Chronic kidney disease (CKD) |

| **TABLE 3. Coding of chronic conditions used in CCI and BEACH MULTIMORBIDITY counts** | |
| --- | --- |
| **Condition** | **Free text terms searched in the EHR diagnosis field** |
| Myocardial infarction (MI) | MI |
|  | AMI |
|  | ACUTE ISCHAEMIC HEART DISEASE |
|  | MYOCARDIAL INFARCTION |
|  | HEART ATTACK |
|  | STEMI |
|  | SUBENDOCARDIAL INFARCT |
|  | ANGIOGRAM-MILD HEART DISEASE |
| PERIPHERAL VASCULAR DISEASE | PVD |
|  | PERIPHERAL VASCULAR DISEASE |
|  | DIABETES VASCULAR DISEASE |
|  | ARTERITIS-DIABETES MELLITUS |
|  | BUERGER’S DISEASE |
|  | OBLITERATIVE VASCULAR DISEASE |
|  | DIABETIC ENDARTERITIS |
|  | THROMBANGITIS OBLITERANS |
|  | ARTERIOSCLEROSIS OBLITERANS |
|  | DIABETES WITH VASCULAR CHANGES |
|  | OCCLUSIVE VASCULAR DISEASE |
|  | PERIPHERAL ARTERIAL DISEASE |
| CVA/TIA | CEREBRAL HAEMORRHAGE |
|  | CEREBRAL INFARCTION |
|  | CEREBROVASCULAR ACCIDENT |
|  | CVA |
|  | HAEMORRHAGE-INTRACEREBRAL |
|  | STROKE |
|  | INTRACEREBRAL BLEED |
|  | INTRACRANIAL HAEMORRHAGE |
|  | LACUNAR INFARCT |
|  | MIGRAINOUS STROKE |
| CONNECTIVE TISSUE DISEASE | RHEUMATOID ARTHRITIS |
|  | POLYMYALGIA RHEUMATICA |
|  | RA |
|  | SLE |
|  | SCLERODERMA |
|  | LUPUS |
|  | SLE |
|  | SYSTEMIC LUPUS ERYTHEMATOSUS |
|  | POLYMYOSITIS |
|  | DERMATOMYOSITIS |
| PEPTIC ULCER DISEASE | PEPTIC ULCER |
|  | ULCERATIVE REFLUX DISEASE |
|  | GASTRIC ULCER |
|  | STOMACH ULCER |
|  | GORD WITH ULCERATION |
| LIVER DISEASE | CHRONIC HEPATITIS |
|  | CIRRHOSIS |
|  | FATTY LIVER |
|  | LIVER FAILURE |
|  | LIVER DISEASE |
|  | ALCHOLIC LIVER |
|  | LIVER DYSFUNCTION |
|  | LIVER DAMAGE |
|  | NASH |
|  | NAFLD |
|  | NON-SPECIFIC HEPATITIS |
|  | HEPATITIS- COLLAGEN DISEASE |
|  | CHOLESTATIC HEPATITIS |
|  | AUTOIMMUNE HEPATITIS |
|  | DRUG INDUCED HEPATITIS |
| HEMIPLEGIA | HEMIPLEGIA |
| LEUKAEMIA | LEUKAEMIA |
| LYMPHOMA | LYPHOMA |
| MALIGNANT TUMOUR | CANCER |
|  | MALIG |
|  | CARCINO |
|  | METAST |
|  | (Note: skin cancer terms excluded, pre-cancer terms excluded, negative cancer markers excluded, malignant hyperthermia excluded, lentigo maligna excluded) |
| HIV/AIDS | HIV  AIDS  IMMUNODEFICIENCY VIRUS |
| HYPERTENSION | HYPERTENSION |
|  | HT |
|  | HIGH BLOOD PRESSURE |
|  | HIGH BP |
|  | H/T |
|  | HYPERTENSIVE |
|  | HTN |
|  | BP HIGH |
|  | LABILE BP |
|  | BP LABILE |
|  | LABILE BLOOD PRESSURE |
|  | BLOOD PRESSURE LABILE |
|  | HBP |
|  | ELEVATED BLOOD PRESSURE |
|  | RAISED BLOOD PRESSURE |
|  | ANTIHYPERTENSIVE AGENT |
|  | HBPM |
| DIABETES MELLITUS | T2DM |
|  | DIAB |
|  | NIDDM |
|  | MELLITUS |
|  | KETOAC |
|  | DKA |
|  | OSMOLAR |
|  | INSULIN |
| LIPID DISORDER | HYPERLIP |
|  | HYPERCHOL |
|  | HYPERTRIG |
|  | HIGH CHOL |
|  | HYPERLIPOPROTEIN |
|  | HIGH LIPID |
|  | DYSLIP |
| DYSPEPSIA/OESOPHAGEAL DISEASE | GOR |
|  | HH |
|  | HEARTBURN |
|  | DYSPEPSIA |
|  | REFLUX |
|  | BARRETT |
|  | INDIGEST |
|  | PETIC ULCER |
|  | HYPERACIDITY |
|  | SLIDING HERNIA |
|  | REGURGITATION |
|  | OESOPHAGITIS |
|  | HIATUS HERNIA |
|  | EPIGASTRIC PAIN |
|  | BELCHING |
|  | HELICOBACTER PYLORI |
| ASTHMA | ASTHMA |
|  | SAMTER |
|  | WHEEZY BRONCHITIS |
|  | ASTHMATICUS |
|  | ASTHMOID |
| ATRIAL FIBRILLATION | AF |
|  | A/F |
|  | ATRIAL FIB |
|  | A.F. |
|  | A FIB |
| MALIGNANT NEOPLASMS OF THE SKIN | SCC |
|  | BCC |
|  | SQUAMOUS CELL |
|  | BASAL CELL |
|  | MELANOM |
| OSTEOPOROSIS | OSTEOPOROSIS |
|  | OSTEOPOROTIC |
| Hypothyroidism/myxoedema | HYPOTHYROID |
|  | MYXEDEMA |
|  | HASHIMOTOS |
|  | THYROIDITIS |
| ISCHAEMIC HEART DISEASE (IHD) | AMI |
|  | MI |
|  | ACUTE MYOCARDIAL INFARCTION |
|  | MYOCARDIAL INFARCTION |
|  | HEART ATTACK |
|  | ACUTE ISCHAEMIC HEART DISEASE |
|  | ISCHAEMIC HEART DISEASE |
|  | ANGIOGRAM-MILD HEART DISEASE |
|  | SUBENDOCARDIAL INFARCT |
|  | SUBENDOCARDIAL MYOCARDIAL INFARCT |
|  | STEMI |
|  | NSTEMI |
|  | ANGINA |
|  | CAD |
|  | IHD |
|  | CABG |
|  | ANGIOPLASTY |
|  | ATHEROLSCLEROTIC HEART DISEASE |
|  | CORONARY HEART DISEASE |
|  | HEART DISEASE |
|  | PREINFARCTION SYNDROME |
|  | PTA |
|  | CORONARY INSUFFICIENCY |
|  | CORONARY OCCLUSION |
|  | CORONARY ARTERY BLOCK |
|  | ISCHAEMIC VASCULAR DISEASE |
|  | OCCLUSION, CORONARY |
|  | OCCLUSION – CORONARY |
|  | OBSTRUCT & STENT & AORTA |
|  | OBSTRUCT & BYPASS & BILIARY |
|  | CARDIAC ARREST |
| CHRONIC OBSTRUCTIVE PULMONARY DISEASE (COPD) | COPD |
|  | COAD |
|  | EMPHYSEMA |
|  | EMPHYSEMATOUS |
|  | CHRONIC AIRWAY/S LIMITATION |
|  | CHRONIC BRONCHITIS |
|  | CHRONIC OBSTRUCTIVE AIRWAY |
|  | CHRONIC OBSTRUCTIVE PULMONARY |
|  | BRONCHITIS- CHRONIC |
|  | CHRONIC AIRWAYS DISEASE |
|  | CHRONIC AIRFLOW LIMITATION |
|  | CHRONIC AIRWAY/S INFECTION |
|  | CAL |
|  | BRONCHITIS CHRONIC |
| GOUT | GOUT |
| MIGRAINE | MIGRAINES |
|  | MIGRATORY |
| CONGESTIVE HEART FAILURE | HEART FAILURE |
|  | CARDIAC FAILURE |
|  | CCF |
|  | CHF |
|  | COR PULMONAE |
|  | LHF |
|  | RHF |
|  | LVF |
|  | RVF |
|  | CARDIAC DYSFUNCTION |
|  | CARDIOMYOPATHY |
|  | VENTRICULAR FAILURE |
|  | VENTRICULAR DIASTOLIC DYSFUNCTION |
| CHRONIC PAIN | HEADACHE |
|  | PLANTAR FASCITIS |
|  | CARPAL TUNNEL |
|  | RHEUMATOID ARTHRITIS |
|  | DYSARTHROSIS |
|  | SHINGLES |
|  | NEURALGIA |
|  | COMPRESSED NERVE |
|  | SPONDYLOSIS |
|  | BACK PAIN |
|  | BACK PROBLEM |
|  | NECK PAIN |
|  | NECK PROBLEM |
|  | THORACIC PAIN |
|  | THORACIC PROBLEM |
|  | SCIATICA |
|  | BULGING DISC |
| DEMENTIA | DEMENTIA |
|  | DEMENTED STATE |
|  | DEMENTING ILLNESS |
|  | DEMENTIO VASCULAR |
|  | ALZHEIMERS |
| CHRONIC KIDNEY DISEASE | CHRONIC KIDNEY DISEASE |
|  | CHRONIC RENAL DISEASE |
|  | CKD |
|  | RENAL IMPAIRMENT |
|  | DIALYSIS |
|  | KIDNEY DISEASE |
|  | KIDNEY IMPAIRMENT |
|  | KIDNEY DAMAGE |
|  | KIDNEY FAILURE |
|  | KIDNEY INSUFFICIENCY |
|  | KIDNEY END STAGE |
|  | RENAL DISEASE |
|  | RENAL IMPAIRMENT |
|  | RENAL DAMAGE |
|  | RENAL FAILURE |
|  | RENAL INSUFFICIENCY |
|  | RENAL END STAGE |
|  | URAEMIA |
|  | CAPD |
|  | CRF |
|  | PERITONEAL CATHETER |

| **TABLE 4. List of mental health conditions and coding of these conditions** | |
| --- | --- |
| **Condition** | **Free text terms searched in EHR diagnosis field** |
| DEPRESSION | DEPRESSION |
|  | DEPRESS MOOD |
|  | DEPRESSED |
|  | DEPRESSIVE |
|  | MELANCHOL |
| Anxiety | GAD |
|  | ANXIETY |
|  | ANXIOUS |
|  | ANXIOLYTIC |
|  | ANTIANXIETY AGENT PRESCRIPTION |
|  | GENERALISED ANXIETY DISORDER |
| POST-TRAUMATIC STRESS DISORDER | POST TRAUMATIC STRESS DISORDER |
|  | POST-TRAUMATIC |
|  | PTSD |
| OBSESSIVE COMPULSIVE DISORDER | OBSESSIVE COMPULSIVE DISORDER |
|  | OBSESSIVE |
|  | OCD |
| ANOREXIA | ANOREXIA |
| BULIMIA | BULIMIA |
| BI-POLAR | BIPOLAR |
|  | BI-POLAR |
| SCHIZOPHRENIA | SCHIZOPHRENIA |
|  | ADJUSTMENT DISORDER |
| DISSOCIATIVE DISORDERS AND OTHER | ACUTE STRESS |
|  | NEUROTIC DEPRESSION |
|  | PHOBIA |
|  | PANIC |
|  | NERVOUS BREAKDOWN |
|  | STRESS DISORDER |
|  | PSYCHOGENIC |
|  | AGORAPHOBIA |
|  | IRRATIONAL FEAR |
|  | PERSONALITY DISORDER |

| **table 5. List of past knee surgeries** |  |
| --- | --- |
| Arthroscopy | Meniscus repair |
| Open reduction knee | Menisectomy |
| Open repair knee | Lateral release |
| Knee reconstruction | Anterior cruciate ligament repair (ACL repair) |
| Cruciate ligament repair | Medial collateral ligament repair (MCL repair) |
| Clean out knee | Osteotomy knee |
| Debridement knee | Knee chondroplasty |
| Fracture tibial plateau with screws | Fracture tibial plateau with repair |
| Supracondylar fracture femur pin | Avulsion fracture femoral condyle |
| Periprosthetic fracture femoral condyle | Arthrotomy |

| **TABLE 6. List of OA medications by ATC code** | |
| --- | --- |
| **ATC code** | **Drug name** |
| H02AB01 | [betamethasone](https://www.whocc.no/atc_ddd_index/?code=H02AB01) |
| H02AB02 | [dexamethasone](https://www.whocc.no/atc_ddd_index/?code=H02AB02) |
| H02AB04 | [methylprednisolone](https://www.whocc.no/atc_ddd_index/?code=H02AB04) |
| H02AB06 | [prednisolone](https://www.whocc.no/atc_ddd_index/?code=H02AB06) |
| H02AB07 | [prednisone](https://www.whocc.no/atc_ddd_index/?code=H02AB07) |
| H02AB08 | [triamcinolone](https://www.whocc.no/atc_ddd_index/?code=H02AB08) |
| H02AB09 | [hydrocortisone](https://www.whocc.no/atc_ddd_index/?code=H02AB09) |
| H02AB10 | [cortisone](https://www.whocc.no/atc_ddd_index/?code=H02AB10) |
| H02AB15 | [meprednisone](https://www.whocc.no/atc_ddd_index/?code=H02AB15) |
| H02BX01 | [methylprednisolone, combinations](https://www.whocc.no/atc_ddd_index/?code=H02BX01) |
| M01AB01 | [indometacin](https://www.whocc.no/atc_ddd_index/?code=M01AB01) |
| M01AB02 | [sulindac](https://www.whocc.no/atc_ddd_index/?code=M01AB02) |
| M01AB05 | [diclofenac](https://www.whocc.no/atc_ddd_index/?code=M01AB05) |
| M01AB15 | [ketorolac](https://www.whocc.no/atc_ddd_index/?code=M01AB15) |
| M01AB51 | [indometacin, combinations](https://www.whocc.no/atc_ddd_index/?code=M01AB51) |
| M01AB55 | [diclofenac, combinations](https://www.whocc.no/atc_ddd_index/?code=M01AB55) |
| M01AC01 | [piroxicam](https://www.whocc.no/atc_ddd_index/?code=M01AC01) |
| M01AC06 | [meloxicam](https://www.whocc.no/atc_ddd_index/?code=M01AC06) |
| [M01AC56](https://www.whocc.no/atc_ddd_index/?code=M01AC56) | meloxicam, combinations |
| M01AE01 | [ibuprofen](https://www.whocc.no/atc_ddd_index/?code=M01AE01) |
| M01AE02 | [naproxen](https://www.whocc.no/atc_ddd_index/?code=M01AE02) |
| M01AE03 | [ketoprofen](https://www.whocc.no/atc_ddd_index/?code=M01AE03) |
| M01AE14 | [dexibuprofen](https://www.whocc.no/atc_ddd_index/?code=M01AE14) |
| M01AE17 | [dexketoprofen](https://www.whocc.no/atc_ddd_index/?code=M01AE17) |
| M01AE51 | [ibuprofen, combinations](https://www.whocc.no/atc_ddd_index/?code=M01AE51) |
| M01AE52 | [naproxen and esomeprazole](https://www.whocc.no/atc_ddd_index/?code=M01AE52) |
| M01AE53 | [ketoprofen, combinations](https://www.whocc.no/atc_ddd_index/?code=M01AE53) |
| M01AE56 | [naproxen and misoprostol](https://www.whocc.no/atc_ddd_index/?code=M01AE56) |
| M01AG01 | [mefenamic acid](https://www.whocc.no/atc_ddd_index/?code=M01AG01) |
| M01AH01 | [celecoxib](https://www.whocc.no/atc_ddd_index/?code=M01AH01) |
| M01AH02 | rofecoxib |
| M01AH04 | [parecoxib](https://www.whocc.no/atc_ddd_index/?code=M01AH04) |
| [M01AH05](https://www.whocc.no/atc_ddd_index/?code=M01AH05) | etoricoxib |
| [M01AX05](https://www.whocc.no/atc_ddd_index/?code=M01AX05) | glucosamine |
| M01AX25 | [chondroitin sulfate](https://www.whocc.no/atc_ddd_index/?code=M01AX25) |
| M01BA03 | [acetylsalicylic acid and corticosteroids](https://www.whocc.no/atc_ddd_index/?code=M01BA03) |
| M02AA07 | [piroxicam](https://www.whocc.no/atc_ddd_index/?code=M02AA07) |
| M02AA10 | [ketoprofen](https://www.whocc.no/atc_ddd_index/?code=M02AA10) |
| M02AA12 | [naproxen](https://www.whocc.no/atc_ddd_index/?code=M02AA12) |
| M02AA13 | [ibuprofen](https://www.whocc.no/atc_ddd_index/?code=M02AA13) |
| M02AA15 | [diclofenac](https://www.whocc.no/atc_ddd_index/?code=M02AA15) |
| M02AA23 | [indometacin](https://www.whocc.no/atc_ddd_index/?code=M02AA23) |
| M02AA27 | [dexketoprofen](https://www.whocc.no/atc_ddd_index/?code=M02AA27) |
| M02AA28 | [piketoprofen](https://www.whocc.no/atc_ddd_index/?code=M02AA28) |
| M02AB | [capsaicin and similar agents](https://www.whocc.no/atc_ddd_index/?code=M02AB) |
| M02AB01 | [capsaicin](https://www.whocc.no/atc_ddd_index/?code=M02AB01) |
| M02AB02 | [zucapsaicin](https://www.whocc.no/atc_ddd_index/?code=M02AB02) |
| M09AX01 | [hyaluronic acid](https://www.whocc.no/atc_ddd_index/?code=M09AX01) |
| N01BX04 | [capsaicin](https://www.whocc.no/atc_ddd_index/?code=N01BX04) |
| N02AA01 | [morphine](https://www.whocc.no/atc_ddd_index/?code=N02AA01&showdescription=yes) |
| N02AA03 | [hydromorphone](https://www.whocc.no/atc_ddd_index/?code=N02AA03&showdescription=yes) |
| N02AA05 | [oxycodone](https://www.whocc.no/atc_ddd_index/?code=N02AA05&showdescription=yes) |
| N02AA08 | [dihydrocodeine](https://www.whocc.no/atc_ddd_index/?code=N02AA08&showdescription=yes) |
| N02AA51 | [morphine, combinations](https://www.whocc.no/atc_ddd_index/?code=N02AA51&showdescription=yes) |
| N02AA53 | [hydromorphone and naloxone](https://www.whocc.no/atc_ddd_index/?code=N02AA53&showdescription=yes) |
| N02AA55 | [oxycodone and naloxone](https://www.whocc.no/atc_ddd_index/?code=N02AA55&showdescription=yes) |
| N02AA56 | [oxycodone and naltrexone](https://www.whocc.no/atc_ddd_index/?code=N02AA56&showdescription=yes) |
| N02AA58 | [dihydrocodeine, combinations](https://www.whocc.no/atc_ddd_index/?code=N02AA58&showdescription=yes) |
| N02AA59 | [codeine, combinations excl. psycholeptics](https://www.whocc.no/atc_ddd_index/?code=N02AA59&showdescription=yes) |
| N02AA79 | [codeine, combinations with psycholeptics](https://www.whocc.no/atc_ddd_index/?code=N02AA79&showdescription=yes) |
| N02AB02 | [pethidine](https://www.whocc.no/atc_ddd_index/?code=N02AB02&showdescription=yes) |
| N02AB03 | [fentanyl](https://www.whocc.no/atc_ddd_index/?code=N02AB03&showdescription=yes) |
| N02AB52 | [pethidine, combinations excl. psycholeptics](https://www.whocc.no/atc_ddd_index/?code=N02AB52&showdescription=yes) |
| N02AB72 | [pethidine, combinations with psycholeptics](https://www.whocc.no/atc_ddd_index/?code=N02AB72&showdescription=yes) |
| N02AC04 | [dextropropoxyphene](https://www.whocc.no/atc_ddd_index/?code=N02AC04&showdescription=yes) |
| N02AC52 | [methadone, combinations excl. psycholeptics](https://www.whocc.no/atc_ddd_index/?code=N02AC52&showdescription=yes) |
| N02AC54 | [dextropropoxyphene, combinations excl. psycholeptics](https://www.whocc.no/atc_ddd_index/?code=N02AC54&showdescription=yes) |
| N02AC74 | [dextropropoxyphene, combinations with psycholeptics](https://www.whocc.no/atc_ddd_index/?code=N02AC74&showdescription=yes) |
| N02AE01 | [buprenorphine](https://www.whocc.no/atc_ddd_index/?code=N02AE01&showdescription=yes) |
| N02AG01 | [morphine and antispasmodics](https://www.whocc.no/atc_ddd_index/?code=N02AG01&showdescription=yes) |
| N02AG03 | [pethidine and antispasmodics](https://www.whocc.no/atc_ddd_index/?code=N02AG03&showdescription=yes) |
| N02AG04 | [hydromorphone and antispasmodics](https://www.whocc.no/atc_ddd_index/?code=N02AG04&showdescription=yes) |
| N02AJ01 | [dihydrocodeine and paracetamol](https://www.whocc.no/atc_ddd_index/?code=N02AJ01) |
| N02AJ02 | [dihydrocodeine and acetylsalicylic acid](https://www.whocc.no/atc_ddd_index/?code=N02AJ02&showdescription=yes) |
| N02AJ03 | [dihydrocodeine and other non-opioid analgesics](https://www.whocc.no/atc_ddd_index/?code=N02AJ03&showdescription=yes) |
| N02AJ06 | [codeine and paracetamol](https://www.whocc.no/atc_ddd_index/?code=N02AJ06) |
| N02AJ07 | [codeine and acetylsalicylic acid](https://www.whocc.no/atc_ddd_index/?code=N02AJ07&showdescription=yes) |
| N02AJ08 | [codeine and ibuprofen](https://www.whocc.no/atc_ddd_index/?code=N02AJ08) |
| N02AJ09 | [codeine and other non-opioid analgesics](https://www.whocc.no/atc_ddd_index/?code=N02AJ09&showdescription=yes) |
| N02AJ13 | [tramadol and paracetamol](https://www.whocc.no/atc_ddd_index/?code=N02AJ13) |
| N02AJ14 | [tramadol and dexketoprofen](https://www.whocc.no/atc_ddd_index/?code=N02AJ14) |
| N02AJ15 | [tramadol and other non-opioid analgesics](https://www.whocc.no/atc_ddd_index/?code=N02AJ15&showdescription=yes) |
| N02AJ17 | [oxycodone and paracetamol](https://www.whocc.no/atc_ddd_index/?code=N02AJ17) |
| N02AJ18 | [oxycodone and acetylsalicylic acid](https://www.whocc.no/atc_ddd_index/?code=N02AJ18&showdescription=yes) |
| N02AJ19 | [oxycodone and ibuprofen](https://www.whocc.no/atc_ddd_index/?code=N02AJ19) |
| N02AX02 | [tramadol](https://www.whocc.no/atc_ddd_index/?code=N02AX02&showdescription=yes) |
| N02AX06 | [tapentadol](https://www.whocc.no/atc_ddd_index/?code=N02AX06&showdescription=yes) |
| N02BA01 | [acetylsalicylic acid](https://www.whocc.no/atc_ddd_index/?code=N02BA01) |
| N02BE01 | [paracetamol](https://www.whocc.no/atc_ddd_index/?code=N02BE01) |
| [N06AX21](https://www.whocc.no/atc_ddd_index/?code=N06AX21) | duloxetine |
