# Supplementary material for: Assessing the suitability of general practice electronic health records for clinical prediction model development: a data quality assessment
Source: BMC Med Inform Decis Mak. 2021 Oct 30;21:297. doi: 10.1186/s12911-021-01669-6 (PMC8557028; doi:10.1186/s12911-021-01669-6)
Supplement: Supplementary file 2 — Additional file 2: Data linkage methodology. [file 12911_2021_1669_MOESM2_ESM.docx]

**Additional file 2: Data Linkage Methodology**

The NPS MedicineWise EHR data was de-identified at time of data extraction from the various general practice clinics. During the de-identification process, each patient was allocated up to four unique encryption keys known as hashes from the GRHANITE software system (27). Hashes were based on patient surname, first name, date of birth, year of birth, gender, postcode and digits 5-9 of Medicare number. To link the de-identified EHR data with the AOANJRR, the data linkage service provider, BioGrid Australia, created hashes for each patient in the AOANJRR using the GRHANITE software system. The two datasets were then linked through deterministic linkage based on these hashes. BioGrid then assigned unique subject identifiers (USIs) to all patients and removed patient hashes prior to sending the data to our research team. For patients in the AOANJRR, USIs were derived from their first name, middle name, last name, date of birth, gender and digits 5-9 of their Medicare number. For patients with unlinked EHRs, USIs were generated using the GRHANITE software system. A similar approach was used to link the EHR data with the NDI. However, AIHW created hashes for patients in the NDI using GRHANITE and BioGrid assigned USIs to this dataset.
