# Supplementary figures and images for: Assessing the suitability of general practice electronic health records for clinical prediction model development: a data quality assessment
Source: BMC Med Inform Decis Mak. 2021 Oct 30;21:297. doi: 10.1186/s12911-021-01669-6 (PMC8557028; doi:10.1186/s12911-021-01669-6)

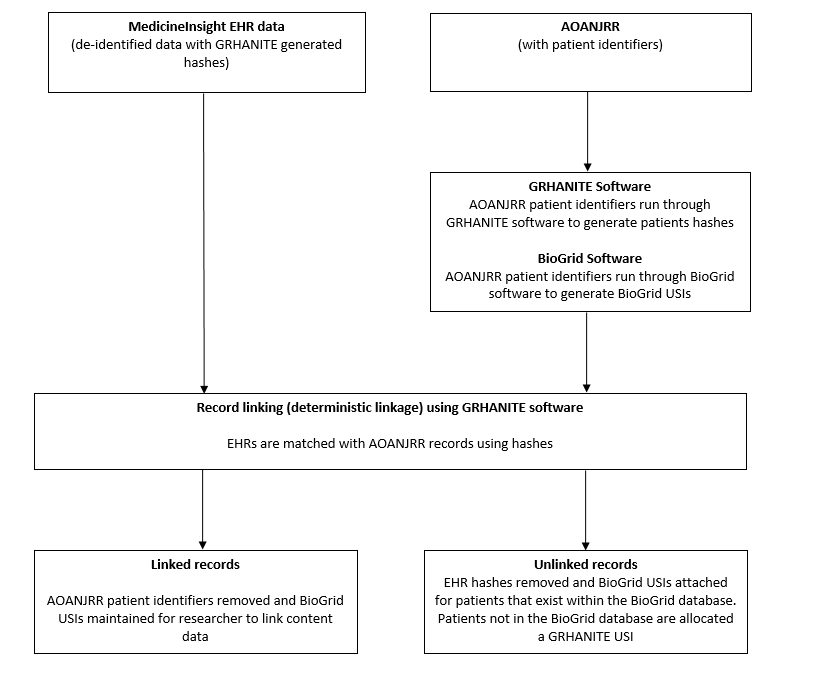

Supplement: Supplementary file 3 — Additional file 3: Methodology for linkage with AOANJRR. [file 12911_2021_1669_MOESM3_ESM.png]

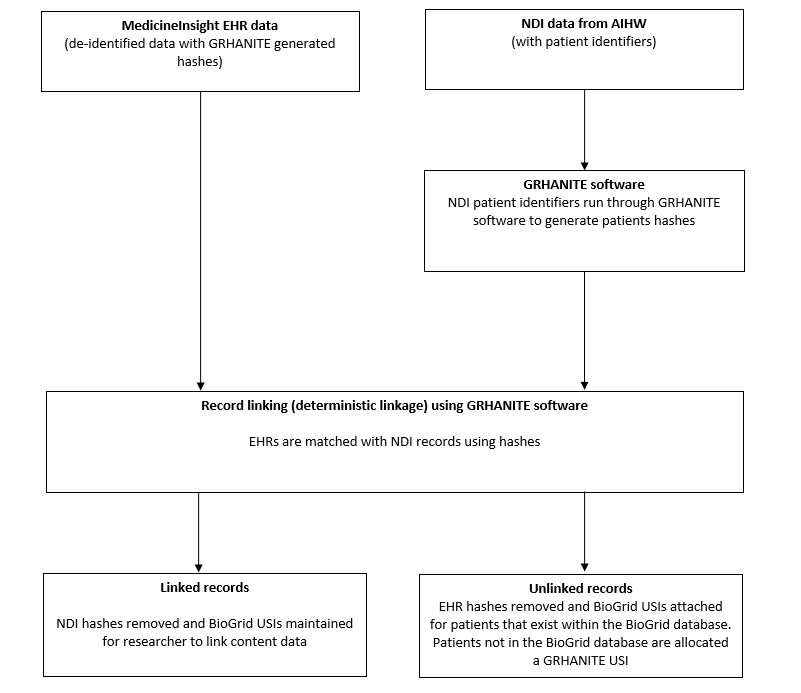

Supplement: Supplementary file 4 — Additional file 4: Methodology for linkage with NDI. [file 12911_2021_1669_MOESM4_ESM.png]
