# Supplementary material for: Assessing the suitability of general practice electronic health records for clinical prediction model development: a data quality assessment
Source: BMC Med Inform Decis Mak. 2021 Oct 30;21:297. doi: 10.1186/s12911-021-01669-6 (PMC8557028; doi:10.1186/s12911-021-01669-6)
Supplement: Supplementary file 5 — Additional file 5: Implausible data entry definitions. [file 12911_2021_1669_MOESM5_ESM.docx]

**Additional file 5: Implausible data entry definitions**

| **Variable** | **Definition of implausible data entry** |
| --- | --- |
| Age at study start | Year of birth beyond data extraction end date of 31^st^ December 2017 |
| BMI | Less than 12 kg/m^2^ or greater than 90 kg/m^2^ |
| Previous/contralateral TKR | Before 01/01/1960 or year of surgery was before year of birth |
| Any past knee surgery |  |
| Total knee replacement |  |
| Death | Before year of birth |
