# Supplementary material for: Assessing the suitability of general practice electronic health records for clinical prediction model development: a data quality assessment
Source: BMC Med Inform Decis Mak. 2021 Oct 30;21:297. doi: 10.1186/s12911-021-01669-6 (PMC8557028; doi:10.1186/s12911-021-01669-6)
Supplement: Supplementary file 6 — Additional file 6: Coding of Australian Bureau of Statistics National Health Survey Chronic Conditions. [file 12911_2021_1669_MOESM6_ESM.docx]

**Additional file 6. Coding of Australian Bureau of Statistics National Health Survey chronic conditions**

| **CONDITION** | **NATIONAL HEALTH SURVEY CONDITION LEVEL CODE (15)** |
| --- | --- |
| HYPERTENSION | 19392 HYPERTENSIVE DISEASE |
| LIPID DISORDER | 14693 HIGH CHOLESTEROL |
| ISCHAEMIC HEART DISEASE | 19117 ANGINA  19383 HEART ATTACK  19382 OTHER ISCHAEMIC HEART DISEASE |
| DEPRESSION | 15907 DEPRESION |
| Anxiety and other neurotic, stress related and somatoform disorders | 15542 ANXIETY DISORDERS  15543 PANIC DISORDERS/PANIC ATTACKS  15548 PHOBIC ANXIETY DISORDERS  15551 POST TRAUMATIC STRESS DISORDER  15556 OTHER MOOD (AFFECTIVE) DISORDERS  15914 OBSESSIVE COMPULSIVE DISORDER  15089 FEELING ANXIOUS, NERVOUS OR TENSE |
| ASTHMA | 20597 ASTHMA |
| DIABETES MELLITUS | 14688 DIABETES MELLITUS- TYPE 1  14689 DIABETES MELLITUS- TYPE 2  14690 DIABETES MELLITUS- TYPE UNKNOWN |
| CHRONIC OBSTRUCTIVE PULMONARY DISEASE (COPD) | 20583 BRONCHITIS  20596 EMPHYSEMA |
| METASTATIC SOLID TUMOUR | 12070 MALIGNANT NEOPLASMS – Other than skin and site unknown |
