# Supplementary material for: Assessing the suitability of general practice electronic health records for clinical prediction model development: a data quality assessment
Source: BMC Med Inform Decis Mak. 2021 Oct 30;21:297. doi: 10.1186/s12911-021-01669-6 (PMC8557028; doi:10.1186/s12911-021-01669-6)
Supplement: Supplementary file 7 — Additional file 7: Assessment of the data linkage process. [file 12911_2021_1669_MOESM7_ESM.docx]

**Additional file 7: Assessment of the data linkage process**

We used the checklist derived by Pratt et al (13) (Table 1) to assess the quality of the data linkage process. Patient linkage keys (hashes) could not be generated for a small proportion of AOANJRR and NDI records, 0.02% (n=126) and 0.05% (n=2,051) respectively, and therefore these records could not be linked with the EHR data. Due to limited access to patient data from AOANJRR and NDI, we were unable to compare characteristics of linked and unlinked patients. However, these proportions are so small that it is unlikely that bias has been introduced through the exclusion of these patients from the study.

Approximately 0.7% (n=1,626) of patients with an EHR had links to more than one record in the NDI, missing date of death or an incorrectly recorded date of death. Patients with common names may have links to multiple records in the NDI, and uncertain dates of death can be due to deaths being discovered some time after the event. These patients were excluded from the study since we could not be certain whether they were alive at study baseline. The characteristics of these excluded patients were similar to those included in the study, except for the prescribing of OA medications, 38% in excluded patients vs. 49% in included patients (Additional file 8). Given the small proportion of patients excluded (0.7%) due to uncertain dates of death, it seems unlikely that study conclusions would differ had these patients been included.

| **Table 1. Data linkage checklist** | | | |
| --- | --- | --- | --- |
|  | **NPS MedicineInsight EHR data** | **AOANJRR data** | **NDI data** |
| **Data sources** |  |  |  |
| Type of data | General practice electronic medical records | Registry data provided by hospitals on AOANJRR forms | Catalogue of all death records in Australia from Registrars of Births, Deaths and Marriages. |
| Provenance of data | Earliest record entered in 1990 | 1999 (national from 2002 onwards) | 1980 |
| Population coverage | 8.2% of Australian general practices | National | National |
| **Linkage variables** |  |  |  |
| Variables used for linkage | Patient hashes derived from GRHANITE during data extraction. Four different hashes created for each patient based on first name, last name, date of birth, year of birth, gender, postcode and digits 5-9 of Medicare number. | First name, last name, middle name, gender, date of birth, digits 5-9 of Medicare number used to create three different patient hashes for each patient using GRHANITE software. Linked to EHR data using hashes. | Patient hashes derived from first name, last name, and date of birth using GRHANITE software. Linked to EHR data using hashes. |
| Quality of linkage and non-linkage variables | Results of DQA assessment in Tables 1-3. | Data are validated internally using State and Territory Health Department data with approximately 94% of records validated. | NDI data validated annually against ABS mortality data. Results are not published. |
| Data cleaning or pre-processing of linkage variables | Nil | AOANJRR carry out logic checks and checks for data entry errors on patient identifiers. | Cleaning of name, death dates and address fields. |
| Special handling or curation of linkage variables | N/a | N/a | N/a |
| **Linkage methods** |  |  |  |
| Linkage methods used | N/a | Deterministic using GRHANITE software and patient hashes. | Deterministic using GRHANITE software and patient hashes. |
| Manual review process | N/a | Manual review for cases where a hash could not be generated | No manual review |
| Linkage service provider | N/a | BioGrid Australia | Australian Institute of Health and Welfare |
| **Linkage results** |  |  |  |
| Linkage proportion and results by linkage type | 17.6% (53,669/304,725) of all patients with an EHR were linked with knee replacement data from the AOANJRR (i.e. had undergone TKR during their life) and 6.7% (20,437/304,725) were matched to a death record in the NDI. 0.09% (281/304,725) of patients with an EHR had links to more than one person in the NDI.  12.6% (25,321/201,462) of all eligible patients with an EHR were linked with knee replacement data from the AOANJRR and 9.1% (18,266/201,462) were linked with death data from the NDI. | Could not generate hashes for 0.02% (126/736,916) of patients in the AOANJRR cohort due to unacceptable first or last names (e.g. father, baby, sister etc.). | Could not generate hashes for 0.05% (2,051/4,046,211) of records in the NDI due to missing patient identifying data |
| **Linkage evaluation** |  |  |  |
| Linkage verification | Patient characteristics were similar between those linked with the NDI and included in the study, and those excluded due to uncertain death dates from the NDI (multiple links to NDI or uncertain death date flag). The only exception was prescribing of OA medications, 49% in linked EHRs in study vs. 38% in EHRs excluded (Additional file 8). | Unable to compare characteristics of patients in linked and unlinked (0.02%) AOANJRR cohorts due to limited access to patient socio-demographic and clinical data from the AOANJRR. | Unable to compare characteristics of patients in linked and unlinked (0.05%) NDI cohorts as no access to patient socio-demographic data from the NDI. |
| Linkage validation | Characteristics of patients in study compared with Australian osteoarthritis population using data from the National Health Survey 2014-2015 (see Table 3). Differences in OA medication usage (EHR 34% vs NHS 55%), metastatic solid tumour (EHR 17% vs NHS 26%) and depression (EHR 20% vs 12%). | TKR rate in 2016/2017 calculated from AOANJRR is approximately 229 per 100,000 per year. AIHW report approximately 218 per 100,000 per year (16). | Unable to validate death data due to limited access to other datasets containing this information. |
