# Supplementary material for: Assessing the suitability of general practice electronic health records for clinical prediction model development: a data quality assessment
Source: BMC Med Inform Decis Mak. 2021 Oct 30;21:297. doi: 10.1186/s12911-021-01669-6 (PMC8557028; doi:10.1186/s12911-021-01669-6)
Supplement: Supplementary file 8 — Additional file 8: Comparison of patient characteristics from EHRs linked with NDI and EHRs excluded due to uncertain dates of death in NDI. [file 12911_2021_1669_MOESM8_ESM.docx]

# Additional file 8. Comparison of patient characteristics from EHRs linked with NDI and EHRs excluded due to uncertain dates of death in NDI

|  | **EHRs linked with NDI data^€^**  **(N=18,266)** | **Missing and implausible data entries** | **EHRs excluded due to uncertain NDI data^€^**  **(N=1,576)** | **Missing and implausible data entries** |
| --- | --- | --- | --- | --- |
| **Characteristics** | **n (%)** | **n (%)** | **n (%)** | **n (%)** |
| ***Predictors*** |  |  |  |  |
| Age (years) – mean (SD) | 75.9 (8.7) | 1 (0.006) | 74.9 (10.1) | - |
| BMI – mean (SD) | 29.0 (6.6) | 11,522 (63.1) | 28.7 (6.2) | 1,102 (69.9) |
| Weight gain between early adulthood and middle age – mean (SD) | - | 18,266 (100.0) | - | 1,576 (100.0) |
| Weight early adulthood – mean (SD) | - | 18,266 (100.0) | - | 1,576 (100.0) |
| Weight middle age – mean (SD) | 88.0 (23.1) | 14,326 (78.4) | 86.1 (20.1) | 1,180 (74.9) |
| OA medication prescription/s | 7,240 (49.2) | 3,556 (19.5) | 430 (38.1) | 447 (28.4) |
| Multimorbidity |  |  |  |  |
| CCI count – Median (IQR) | 1 [0,2] | 2,908 (15.9) | 1 [0,2] | 202 (12.8) |
| BEACH count^ - Median (IQR) | 3 [2,5] | 5,624 (30.8) | 3 [2, 5] | 458 (29.1) |
| Combined CCI and BEACH count^ - Median (IQR) | 4 [2,5] | 5,829 (31.9) | 3 [2, 5] | 467 (29.6) |
| Mental health condition | 4,807 (27.7) | 894 (4.9) | 437 (29.0) | 71 (4.5) |
| Previous/contralateral knee replacement | 1,268 (7.0) | 166 (0.9) | 105 (6.7) | 10 (0.6) |
| Any past knee surgery on either knee (excluding TKR) | 312 (1.7) | 45 (0.2) | 34 (2.2) | 6 (0.4) |
| Patient geographical location |  | 101 (0.6) |  | 15 (1.0) |
| Major cities of Australia | 9,217 (50.7) |  | 799 (51.2) |  |
| Inner regional Australia | 6,250 (34.4) |  | 519 (33.3) |  |
| Remote Australia | 2,698 (14.8) |  | 243 (15.6) |  |
| ***Outcome*** |  |  |  |  |
| TKR during study | 327 (1.8) | 1 (0.01) | 46 (2.9) | 12 (0.8) |

Counts and percentages presented unless otherwise indicated. Percentages may not sum to 100% due to rounding.

€Patients who underwent bilateral TKR prior to study baseline (study inclusion criteria iv) have been excluded.

Abbreviations: BMI-body mass index; OA-osteoarthritis; CCI-Charlson Comorbidity Index; IQR- Inter-Quartile Range; BEACH-Bettering the Evaluation and Care of Health; TKR-total knee replacement

^excluding mental health conditions

Notes: BMI includes measurements recorded within one year of study baseline; Early adulthood=18-21 years; Middle age =45-65 years; Patient considered to be on OA medication if estimated to be on medication at study baseline using prescription date and medication strength, dosage and frequency; Patient considered to have chronic condition or undergone past knee surgery if record of this exists prior to study baseline
